# The Index-Based Subgraph Matching Algorithm (ISMA): Fast Subgraph Enumeration in Large Networks Using Optimized Search Trees

**DOI:** 10.1371/journal.pone.0061183

**Published:** 2013-04-19

**Authors:** Sofie Demeyer, Tom Michoel, Jan Fostier, Pieter Audenaert, Mario Pickavet, Piet Demeester

**Affiliations:** 1 Department of Information Technology, Ghent University, Ghent, Belgium; 2 School of Life Sciences - LifeNet, Freiburg Institute of Advanced Studies, Freiburg, Germany; 3 Division of Genetics and Genomics, The Roslin Institute - University of Edinburgh, Midlothian, Scotland, United Kingdom; King Abdullah University of Science and Technology, Saudi Arabia

## Abstract

Subgraph matching algorithms are designed to find all instances of predefined subgraphs in a large graph or network and play an important role in the discovery and analysis of so-called network motifs, subgraph patterns which occur more often than expected by chance. We present the index-based subgraph matching algorithm (ISMA), a novel tree-based algorithm. ISMA realizes a speedup compared to existing algorithms by carefully selecting the order in which the nodes of a query subgraph are investigated. In order to achieve this, we developed a number of data structures and maximally exploited symmetry characteristics of the subgraph. We compared ISMA to a naive recursive tree-based algorithm and to a number of well-known subgraph matching algorithms. Our algorithm outperforms the other algorithms, especially on large networks and with large query subgraphs. An implementation of ISMA in Java is freely available at http://sourceforge.net/projects/isma/.

## Introduction

Over the last decade, network theory has come to play a central role in our understanding of complex systems in fields as diverse as molecular biology, sociology, economics, the internet, and others [1]. The central question in all these fields is to understand behavior at the level of the whole system from the topology of interactions between its individual constituents. In this respect, the existence of *network motifs*, small subgraph patterns which occur more often in a network than expected by chance, has turned out to be one of the defining properties of real-world complex networks, in particular biological networks [2]. Network motifs act as the fundamental information processing units in cellular regulatory networks [3] and they form the building blocks of larger functional modules (also known as network communities) [4]–[6]. The discovery and analysis of network motifs crucially depends on the ability to enumerate all instances of a given query subgraph in a network or graph of interest, a classical problem in pattern recognition [7], that is known to be NP complete [8].

Subgraph matching algorithms are usually classified as either exact algorithms, which require a strict correspondence between the query graph (i.e. the subgraph) and any match in the target graph, or inexact algorithms, where some deformation of the query graph is allowed when searching for a match [7]. Here we are only concerned with exact algorithms. Some of the most well-known algorithms that realize this are the algorithm of Ullmann [9], the VF [10], [11] and the VF2 algorithm [12], [13]. The algorithm of Ullmann uses the adjacency matrix representation of the networks. A number of auxiliary matrices are defined to determine the set of subgraph isomorphisms iteratively. In the VF algorithms, on the other hand, the networks are represented by graphs. A state space representation is used in which each state depicts a (partial) mapping between nodes of both networks. The algorithm recursively builds a network of states by adding to the present states a pair of nodes that can be mapped on each other. After computing a set of candidate pairs, for each pair it is checked whether it meets the feasibility rules. Only then a new state is created. The difference between the VF and the VF2 algorithm is that the exploration of the search space has been improved in the VF2 algorithm to reduce memory requirements. This means that it is faster and can also be applied in larger graphs.

Most of the other exact algorithms typically find subgraph isomorphisms in a database of graphs. To realize the subgraph matching efficiently, a preprocessing step on this database is introduced. Messmer and Bunke [14] proposed a method consisting of building a decision tree from the database of graphs by a form of indexing. This structure can then be used to find all subgraph instances. This preprocessing step has been further optimized by Weber et al. [15]. Another algorithm, the GraphGrep algorithm [16], uses hash-based fingerprinting to index the database of graphs. GIndex [17] makes use of frequent substructures for its indexing. The GADDI algorithm [18] on the other hand deals with larger graphs and uses an indexing based on a neighborhood structure, similar to the TALE algorithm [19]. Another way to deal with exact subgraph matching is to reformulate it as a constraint satisfaction problem and solving it with constraint programming, which is a good approach if there are other constraints that need to be taken into account as well [20], [21].

Motivated by problems in biology, where it is necessary to find subgraph instances in graphs with certain characteristics on the links, which define the type of interaction between cellular components (e.g. protein-protein, protein-DNA or protein phosphorylation, etc.) [6], [22], [23], we developed a novel exact subgraph matching algorithm, which uses a search tree to find all instances of a query subgraph in an edge-colored graph without using an additional, usually time consuming, preprocessing step. The algorithms that resemble our algorithm most are the algorithm of Ullmann [9], the VF [10], [11] and the VF2 algorithm [12], [13]. Note that our problem differs from for example the SAGA algorithm [24] in which the nodes instead of the edges contain certain characteristics.

At the heart of our algorithm are custom designed data structures (for both the network and the algorithm) which provide, at each step in the subgraph matching procedure, rapid indexing of the candidate nodes for inclusion in a subgraph instance. By carefully selecting the order in which the motif nodes (denoted by an index) are investigated, these sets of candidate nodes are kept as small as possible. This allows to cut unfavorable branches in the search tree as soon as possible and leads to a dramatic speedup compared to existing algorithms. In this paper, we present a formal description of the Index-based Subgraph Matching Algorithm (ISMA), the data structures and how symmetries in the query subgraph are dealt with. This paper is organized as follows. After giving a general overview of the problem, together with the definitions of the concepts that are used in this article, a naive recursive algorithm is presented. The weaknesses of this algorithm are then identified and an improved recursive ISMA algorithm is proposed. As iterative algorithms may achieve a performance gain and require less stack space and function call overhead, an iterative version of the ISMA algorithm is presented, for which a number of custom data structures were designed. We also present comparisons to related subgraph matching algorithms using a variety of biological and non-biological networks.

## Methods

### General description

In biological networks, the same set of nodes (typically genes or proteins) can be connected in different ways, representing different physical interaction mechanisms, which may be directed or not [25]. In order to find matches for so-called composite motifs (subgraph patterns with more than one interaction type [22], [23]), the ISMA algorithm is designed to find all occurrences of a given query subgraph in graphs 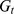 with annotated edges. More precisely, 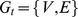 with 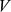 the set of vertices (or nodes) and 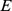 the set of edges (or links), where each link is represented by a triplet 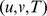 with 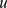 and 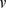 the start and end node respectively, and 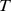 the type of the link. Hence, in contrast to ordinary graphs, the links now also have a type, which identifies a number of characteristics of the link such as whether it is directed or undirected. It should be noted that parallel links are allowed in 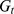 if and only if they are of a different type.

**Figure 1:**
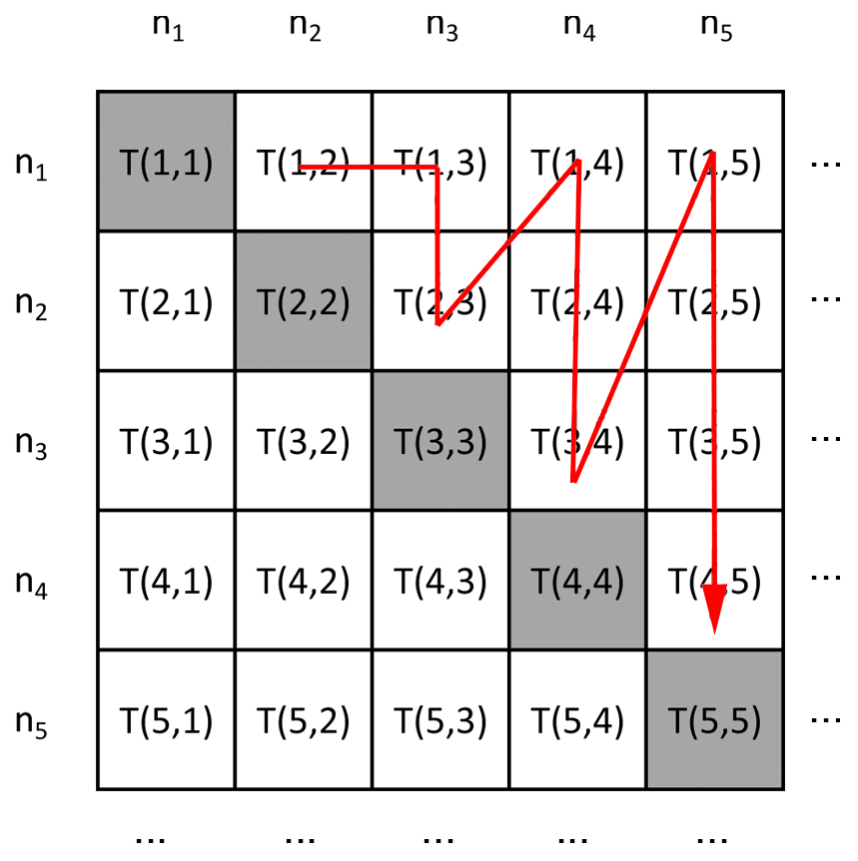
The motif adjacency Matrix. In this article, motifs are denoted by a motif specification which can be deduced from its adjacency matrix as indicated by the red arrow.

A motif or query subgraph 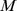 is defined as follows. It is a small graph of 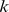 nodes with no (anti-)parallel links. This means that a motif has at most 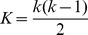 links. The assumptions of no (anti-)parallel links is not a strict condition and the research presented here can easily be extended to motifs with (anti-)parallel links. There are four possible configurations between two nodes 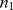 and 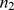: no link, a directed link from 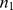 to 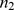, a directed link from 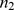 to 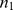, or an undirected link between 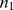 and 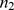. The motif nodes (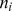) are ordered and can hence be referred to by a unique index 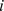. In the remainder of this article, this index will be used to refer to the motif nodes themselves. Motifs are specified by a list of K link types as follows:
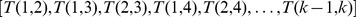
with 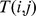 the link type between the 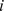-th and the 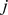-th node of the motif. It is defined that if no link exists between two nodes in a motif the corresponding link type is set to 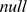 (or ‘0’). This motif specification can easily be deduced from the adjacency matrix of the graph as indicated in figure 1.

**Figure 2:**
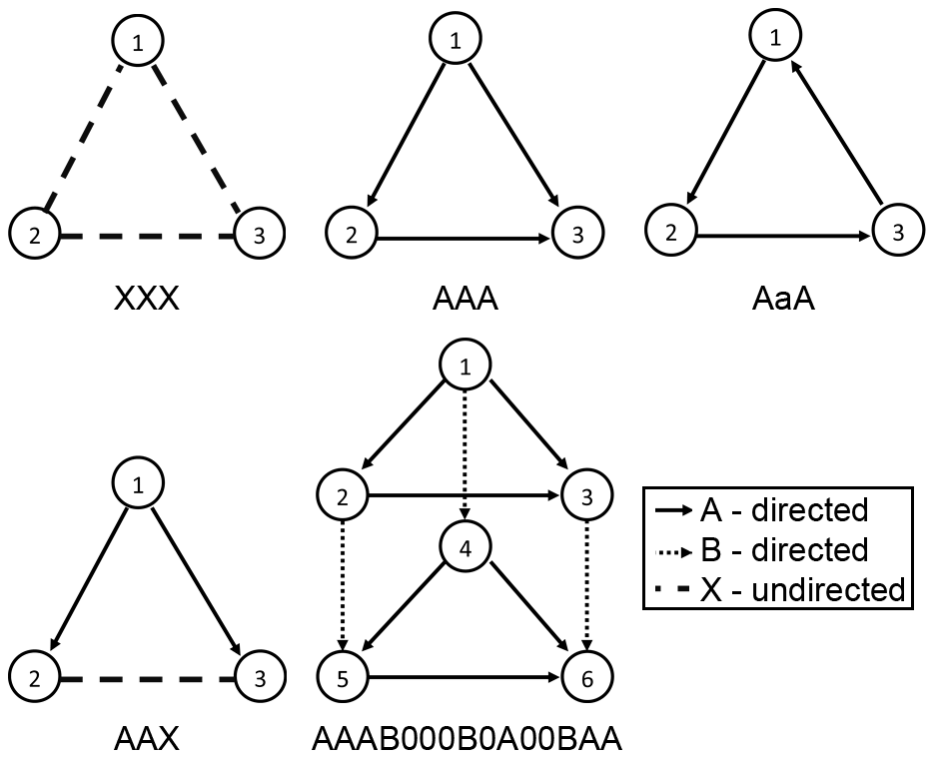
Examples of motifs and their specifications. Here nodes are denoted by their index. Look at for example the motif AAAB000B0A00BAA. Its motif specification can be deduced as follows: a directed link of type 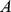 from node 1 to node 2, a directed link of type 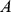 from node 1 to node 3, a directed link of type 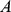 from node 2 to node 3, a directed link of type 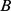 from node 1 to node 4, no link between node 2 and node 4, no link between node 3 and node 4, no link between node 1 and node 5, a directed link of type 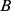 from node 2 to node 5, no link between node 3 and node 5, a directed link of type 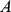 from node 4 to node 5, no link between node 1 and node 6, no link between node 2 and node 6, a directed link of type 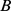 from node 3 to node 6, a directed link of type 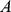 from node 4 to node 6, a directed link of type 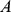 from node 5 to node 6.

Link types may be specified by upper case characters (A, B, etc.). In the case the links are directed, the reverse of a link can be represented by the corresponding lower case character. A number of examples of motifs and their specification are given in figure 2.

### The naive recursive subgraph matching algorithm (RSMA)

In this section, a naive recursive subgraph matching algorithm is described which is implemented in a motif clustering software tool (Cyclus 3D) [26]. We describe this algorithm in detail here, as it will form the basis for the ISMA algorithm. It is a depth first tree search procedure in which the motif nodes are investigated in the order in which they are listed in the specification. This means that the algorithm will first map a network node on the first motif node, then on the second motif node, and so on.

It should be noted that this algorithm resembles the VF2 algorithm. However, it is not completely similar. In the VF2 algorithms first a set of candidate pairs (i.e. a network node and a motif node on which this network node can be mapped) is calculated and subsequently this set is filtered according to the feasibility rules. One of these rules, for example, checks whether the links have the correct attributes (i.e. link types). By a careful design of the network data structure in the ISMA algorithm all candidate nodes are feasible nodes, which means that no additional checking operation is needed.

The pseudocode of the recursive subgraph matching algorithm (RSMA) is given below. The algorithm (i.e. the function findMotifs) takes 3 input parameters: a motif specification mspec, the motif instance instance that tracks which network nodes have been mapped on the motif nodes, and the network 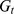. In each recursive call, it is first checked whether the instance is complete, i.e. whether all motif nodes (mn) have network nodes (nn) mapped on them. If this is the case, the motif instance is exported. Otherwise, the next motif node (specified by its index) to be investigated is identified by the function next(). Here, next() returns the smallest index that has not been investigated yet. Subsequently, a set is determined of all network nodes that can be mapped on this motif node. This set contains all network nodes that are connected to the network nodes that were already mapped in the instance by links of the correct type (according to the motif specification). For the first motif node, this set simply consists of all nodes of the network 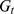. One by one, the network nodes in this set are mapped onto the motif node, after which the findMotifs routine is called recursively. This way, all instances in 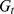 corresponding to the motif specification are enumerated.

### Recursive subgraph matching algorithm

1 findMotifs(mspec, instance, Gt){

2  if(instance is complete){

3   export(instance);

4   return;

5  }

6  mn = next();

7  set = determineSet(instance, mn, Gt);

8  forall(nodes nn in set){

9   instance.put(mn, nn);

10   findMotifs(mspec, instance, Gt);

11   previous();

12   instance.remove(nn);

13  }

14 }

15

16 i = 0;

17 next(){

18  return i++;

19 }

20

21 previous(){

22  i–;

23 }

24

25 determineSet(instance, mn, Gt){

26  if(index =  = 1){

27   return V;

28  }else{

29   sets = EMPTY;

30   forall(nodes ni in instance){

31    motifIndex = instance.getIndex(ni);

32    linkType = mspec.getLinkType(motifIndex, mn);

33    set = ni.getNeighborsofType(linkType);

34    sets.add(set);

35    }

36    return intersection(sets);

37  }

38 }

The RMSA algorithm has some efficiency issues. When the set of candidate network nodes is determined for the first motif node, the complete set of network nodes is returned (line 22). It is possible that a lot of these network nodes don't even have the correct links (according to the motif specification) departing from them and thus are bad candidates to be mapped on the first motif node. This means that the search tree is very broad near the root. It would be better to narrow this down and determine a set of good candidate nodes by checking the types of the links that are departing from the network nodes and only select those nodes that have links of the same type as the links from the first motif node. Moreover, this set of candidate nodes can be further reduced by selecting another motif node to be investigated first. By changing the order in which the motif nodes are investigated (in the next()-function), the sets of candidate network nodes can be kept as small as possible. Smaller sets lead to less branches in the search tree and thus faster calculation times.

### Operation of the ISMA algorithm: an example

In this section, we will sketch the operation of the index-based subgraph matching algorithm by means of an example. In the three following sections, the algorithm is described in full detail.

**Figure 3:**
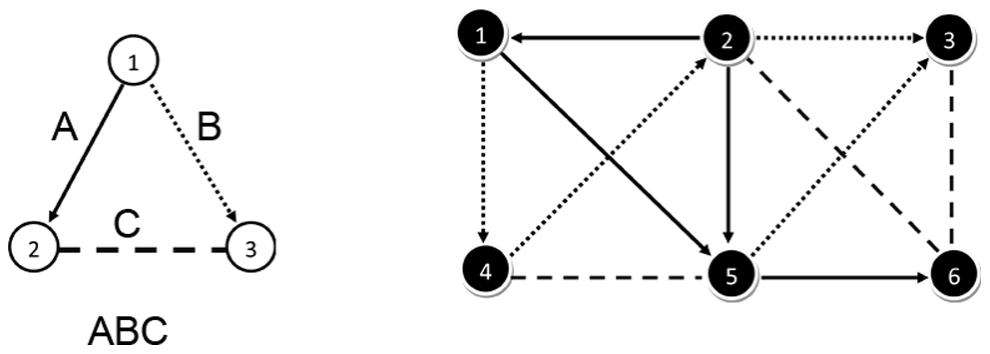
Example motif and network. The motif (left) which is searched for in the example network (right). Links of type A or B are directed; links of type C are undirected.

Suppose we want to find all occurrences of the motif ABC (with link types A and B directed, and link type C undirected) in the network depicted in figure 3. In the initialization phase, the best motif node to be investigated first is determined. As we want to narrow down the search tree, this should be the motif node with the least number of possible network nodes that can be mapped on it. These network nodes are the nodes that have the same types of outgoing links as the motif node. The set of possible network nodes for each motif node can then be determined by calculating the intersection of the sets of start network nodes of the corresponding link types. As calculating these intersections can be quite time-consuming, we opted to calculate the number of start nodes for each link type of the motif and select the motif node from which a link departs of the type with the lowest number of start nodes. This is shown in table 1. In practice, these sets of network (start) nodes are retrieved in constant time since they are stored in the data structure of the network itself (see section on data structures). It should be noted that if some of the links are directed, the occurrences of the reverse links should also be taken into account. In our example, this means that the sets of starting network nodes from the link types a and b also need to be determined. The link type with the lowest cardinality of its corresponding set determines the first motif node, namely the motif node from which a link of this type departs.

**Table 1:** The initialization phase.

link type	{nn}	# nn	link type	{nn}	# nn
A	{1,2,5}	3	a	{1,5,6}	3
B	{1,2,4,5}	4	b	{2,3,4}	3
C	{2,3,4,5,6}	5			

For each link type the set of network nodes is depicted together with its cardinality.

If there are multiple link types with the same cardinality, there are two possibilities: randomly picking one of these link types or actually calculating the sets of possible network nodes that can be mapped on the motif nodes. Here, we will apply the latter and calculate for the concerned motif nodes the intersection of the sets of starting network nodes of the links of which the types are specified in the motif. In our example, we encounter the same cardinality for the link types A, a and b, which correspond to motif nodes 1 (for A), 2 (for a) and 3 (for b). For the motif node 1, the set of possible network nodes is the intersection of the start sets of link type A ({1,2,5}) and link type B ({1,2,4,5}), thus set {1,2,5}. Similarly, for motif node 2 (link types a and C) and motif node 3 (link types b and C) this results in {5,6} and {2,3,4} respectively. From this we can conclude that motif node 2 is the best option to be investigated first as its set of candidate network nodes only has 2 elements, namely network nodes 5 and 6.

We will map one of these nodes, network node 5, on motif node 2. Now it needs to be determined which of the 2 remaining motif nodes (1 or 3) is the best option to be investigated next. To do this, we determine the cardinality of the sets of network nodes that are neighbors of network node 5 according to the correct link types in order to be mapped on the specific motif nodes. For motif node 1 (connected to motif node 2 with link type a) this set of network nodes is {1,2}, while for motif node 3 (connected to motif node 2 with link type C) this set is {4}. As there is only one network node that can be mapped on motif node 3, we will consider this motif node to be investigated next.

Network node 4 is mapped on motif node 3. Now, we need to determine which network nodes can be mapped on the last motif node, namely node 1. This is the intersection of the set of neighbors of network node 5 according to link type a ({1,2}) and the set of neighboring network nodes of node 4 according to link type b ({1}). This results in singleton {1}. We now have a complete instance that can be exported.

As there are no other network nodes that can be mapped on motif node 1 (all nodes of the set {1} have been mapped), and no other network nodes can be mapped on motif node 3 (all nodes of the set {4} have been mapped), we will map the next network node on motif node 2, namely node 6 (from the set {5,6} determined at initialization). Again, it will be determined which of the remaining motif nodes (1 or 3) will be investigated first. For motif node 1, the set of possible network nodes (neighbors of network node 6 according to link type a) is {5}. For motif node 3 (connected to motif node 2 with link type C) this set is {2,3}. Now motif node 1 is the best option to be investigated first as there is only one network node that can be mapped on it. Network node 5 is mapped on motif node 1. To determine which network nodes can be mapped on the last motif node 3, the intersection is calculated between the set of neighbors of network node 6 according to link type C ({2,3}) and the set of neighbors of network node 5 according to link type B ({3}). This results in the singleton {3}. Network node 3 is mapped om motif node 3 and the complete instance can be exported.

There are no more network nodes that can be mapped on motif node 3 (all nodes of the set {3} have been mapped), and no more network nodes that can be mapped on motif node 1 (all nodes of the set {5} have been mapped). Moreover, we iterated over all network nodes that can be mapped on motif node 2 (all nodes of the set {5,6} have been mapped). This means that the algorithm can terminate and has found all instances of the motif ABC in the network. Two motif instances have been found, one with the network nodes 1, 5 and 4 mapped on motif nodes 1, 2 and 3 respectively, and one with the network nodes 5, 6 and 3.

Similar to the naive recursive algorithm, this algorithm is also a depth-first search algorithm. When 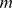 motif nodes have network nodes mapped on them, first all possibilities for the remaining 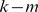 nodes (assuming the motif has 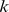 nodes) will be checked, before mapping the next network node on the 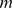-th motif node. As mentioned before, in this algorithm motif nodes are not always investigated in the same order. In the above example, in both instances motif node 2 was investigated first, but for the first instance motif node 3 was the next to be investigated, while for the second instance this was motif node 1. By carefully selecting the order in which the motif nodes are investigated, the sets of network nodes that can be mapped on the motif nodes are kept as small as possible, reducing the number of branches in the search tree.

**Figure 4:**
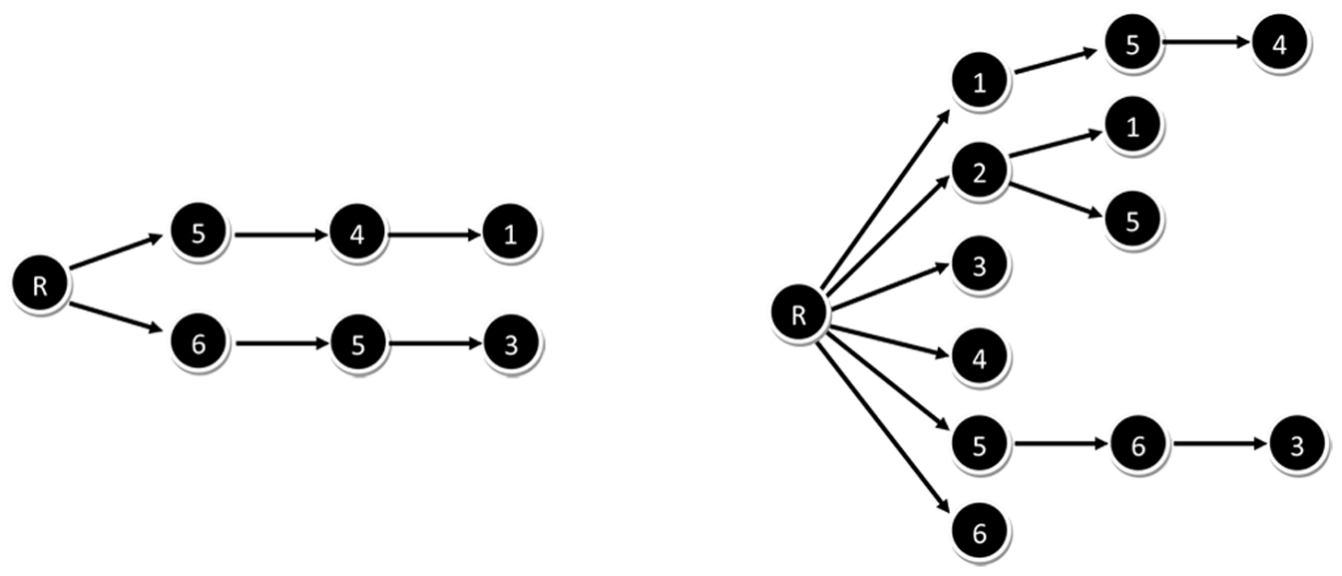
Search tree. The search tree of the ISMA algorithm (left) and the standard recursive algorithm (right) applied to the example network. A search tree indicates which network nodes have been mapped on the motif nodes.

The search trees of both the RSMA and the ISMA algorithm are depicted in figure 4. It can be seen that the ISMA algorithm indeed has a significantly smaller search tree, namely 6 nodes instead of 12.

### The recursive index-based subgraph matching algorithm

Below, the pseudo code of the recursive index-based subgraph matching algorithm is presented. One can see that the main algorithm (i.e. the findMotifs-function) is similar to the one of the naive recursive algorithm. The determineSet-function only differs in the case in which no network nodes have been mapped on the motif nodes yet. Instead of returning the complete set of network nodes, now a set of good candidate network nodes for this motif node is returned. These are the networks nodes that have the same links (or more accurately link types) departing from them as the specific motif node.

The next-function returns the best motif node to be investigated next. This is the motif node that has the smallest set of candidate network nodes, as this leads to a smaller search tree. In the case the motif instance is empty (i.e. no network nodes have been mapped on the motif nodes), we will determine for each link type the number of networks links of this type. The link type with the lowest cardinality determines the best motif node. As stated in the previous section, when multiple link types have the same cardinality (i.e. the boolean variable multiple is true), there are two options: randomly selecting one of these link types or calculating the sets of possible network nodes that can be mapped on the motif nodes. The code depicts the latter one. For each motif node the set of candidate network nodes is determined. These are the network nodes with the same outgoing link types as the motif node. The motif node with the smallest set of candidate nodes is the best option to be investigated first. In the case where some of the motif nodes have network nodes mapped on them, we will determine for each of the unmapped motif nodes the number of neighbors of the mapped network nodes that can be mapped on this motif node. The minimum number then determines which motif node will be investigated next. By always selecting the motif node with the smallest set of candidate network nodes, the number of branches in the search tree is kept as small as possible, leading to faster calculations.

### Recursive ISMA algorithm

1 findMotifs(mspec, instance, Gt){

2  if(instance is complete){

3   export(instance);

4   return;

5  }

6  mn = next(instance, mspec);

7  set = determineSet(instance, mn, Gt, mspec);

8  forall(nodes nn in set){

9   instance.put(mn, nn);

10   findMotifs(mspec, instance, Gt);

11   instance.remove(nn);

12  }

13 }

14

15 next(instance, mspec){

16  min = LARGE NUMBER;

17  if(instance is empty){

18   multiple = FALSE;

19   linkTypes = mspec.getLinkTypes();

20   forall(types t in linkTypes){

21    #NBS = #fGt.getStartNodesofType(t)};

22    if(#NBS<min){

23     min = #NBS;

24     i = t.getStartNode();

25     multiple = FALSE;

26    }else if(#NBS =  = min){

27     multiple = TRUE;

28    }

29   }

30   if(multiple){

31    forall(motif nodes mn){

32     #NBS = #fdetermineSet(instance, mn, *Gt*, mspec)};

33     if(#NBS<min){

34      min = #NBS;

35      i = mn;

36     }

37    }

38   }

39  }else{

40   forall(unmapped motif nodes mn){

41    forall(mapped network nodes nn){

42     #NBS = number of neighbors of nn that can be mapped on mn;

43     if(#NBS<min){

44      min = #NBS;

45      i = mn;

46      }

47    }

48   }

49  }

50  return i;

51 }

52

53 determineSet(instance, mn, Gt, mspec){

54  if(instance is empty){

55   linkTypes = mspec.getLinkTypesFrom(mn);

56   forall(types t in linkTypes){

57    set = Gt.getStartNodesofType(t);

58    sets.add(set);

59   }

60   return intersection(sets);

61  }else{

62   sets = EMPTY;

63   forall(nodes ni in instance){

64    motifIndex = instance.getIndex(ni);

65    linkType = mspec.getLinkType(motifIndex, mn);

66    set = ni.getNeighborsofType(linkType);

67    sets.add(set);

68   }

69   return intersection(sets);

70  }

71 }

### Data structures

As shown in the example and the recursive ISMA algorithm, the execution time of the recursive algorithm can be reduced by an intelligent choice of the order in which the nodes of a motif are investigated. This way, unfavorable branches of the search tree are pruned as soon as possible. Moreover, by an intelligent design of the network data structure an additional speedup can be realized for both the RSMA and the ISMA algorithm.

**Figure 5:**
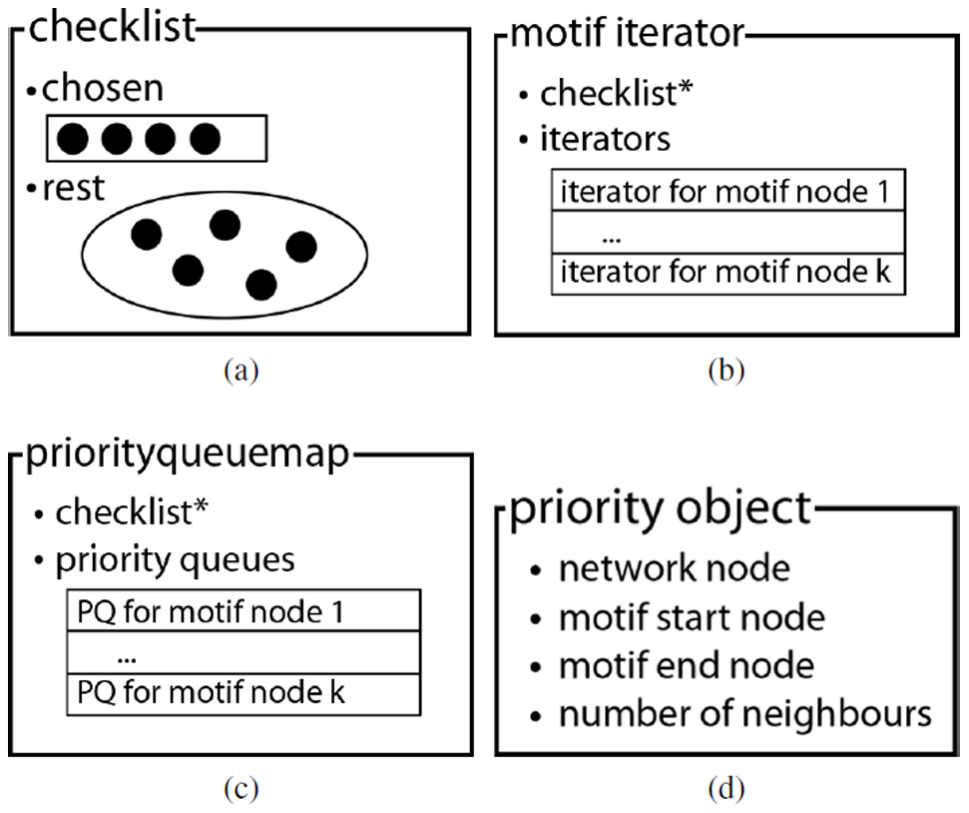
Data Structures. (a) The checklist. In the ISMA algorithm, the circles represent motif nodes (b) The motif iterator. (c) The priority queue map. (d) The priority object. It is assumed that the motif has k nodes.

This section starts with an overview of the optimizations to the network data structure which enable fast retrieval of the network nodes adjacent to links of a certain type. Subsequently, a number of data structures (see figure 5) are presented in order to realize an iterative version of the ISMA algorithm (which is more efficient and requires less stack space). The checklist keeps track of the order in which the motif nodes are investigated. A motif iterator is a collection of iterators that iterate over the possible network nodes for each of the motif nodes. The priority queue map is used in the algorithm to determine which of the motif nodes is the most lucrative to be investigated next. Investigating a motif node here means determining a set of network nodes that can be mapped on it, and adding each of these nodes to the instance one after the other.

**Figure 6:**
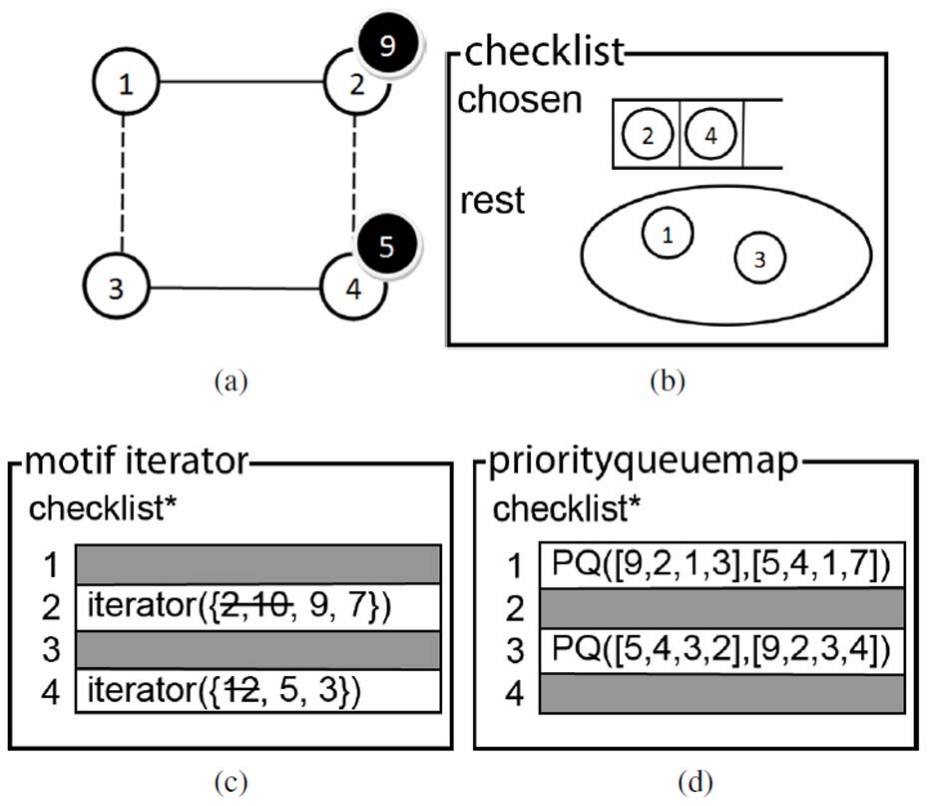
Example of data structures. We are looking for a 4-node motif. In the motif instance (a) network node 9 is mapped on motif node 2 and network node 5 is mapped on motif node 4. These motif nodes were added (in the correct order) to the chosen list of the checklist (b), while the other two motif nodes (1 and 3) remain in the rest set. The motif iterator (c) contains two iterators that are of importance, namely the ones for the motif nodes of the chosen list. These iterate over the possible network nodes that can be mapped on the motif nodes. The priorityqueuemap (d) only contains valuable priority queues for the motif nodes 1 and 3. Each priority queue contains a priorityobject for each network node that is already mapped in the instance.

To better understand the algorithm specific data structures, figure 6 presents an example in which the possible content of the data structures is given for one instant during the execution of the algorithm.

#### Optimizations to the network data structure

The network data structure contains a network (i.e. graph). The main structure consists of a list of nodes. Each of these nodes has a list of neighbors, together with the links connecting them. The data structure has been optimized for two specific operations in the ISMA algorithm: the retrieval of the start nodes of all links of a specific type and the retrieval of all neighbors of a node that are connected to that node with a link of a specific type. This has been accomplished by 2 changes:

- In the network structure a map is added with the link type as key and the set of the start nodes of all links of this link type as value. As the number of link types in a network is relatively small, finding the start nodes of all links of a specific type now only requires a small amount of execution time.
  However, this structure requires some additional memory. In a map, for each link type a set of reference to nodes (i.e. start nodes of links of this type) is stored. Assuming that the network has 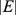 links and that there are 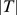 link types, this means that the additional memory needed is equal to 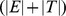 references plus the memory overhead of one map and 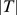 sets.
- For every node a map is kept with the link types as keys and the sets of all neighbors according to this link types as values. This speeds up the operation of finding all neighbors that are connected with a link of a specific type.
  As in ‘non-optimized’ networks nodes also contain references to their neighbors, this structure only needs a small amount of additional memory, namely 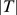 references to the link types plus the memory overhead of one map and 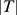 sets.

#### Checklist

A checklist (CL) is a data structure that keeps track of the order in which elements (motif nodes) are chosen from a collection. It contains an ordered list of the chosen elements, together with the set of all elements that have not been chosen yet. It should be noted that in the recursive algorithm, this information is kept in the stack. The check list data structure is illustrated in figure 5a.

In the example (see figure 6b) first motif node 2 was removed from the rest set and added to the chosen list. Subsequently motif node 4 was removed from the rest set and added to the chosen list. Two nodes (1 and 3) remain in the rest set.

Following functions are defined on an checklist:

- numberChecked(): returns the number of chosen elements
- lastChecked(): returns the last element that has been chosen
- check(element): removes element from the rest-set and adds it to the list of chosen elements
- uncheck(): removes the last element from the chosen-list and adds it to the rest-set
- checked(): returns the list of checked (i.e. chosen) elements
- rest(): returns the rest-set

#### Motif iterator

The motif iterator (MI) contains an iterator for each of the motif nodes that iterates over the possible network nodes that can be mapped on this motif node. Additionally, in order to know the order in which the motif nodes have been investigated, it contains a pointer to a checklist. When a motif node has not been investigated yet, the corresponding iterator is set to 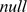. This data structure is depicted graphically in figure 5b.

Whereas for the ‘first’ motif node the iterator stays the same during the complete execution of the algorithm, the iterators for the other motif nodes will change. Every time a motif node is determined as the best to be investigated next, a new iterator (that iterates over the set of possible network nodes that can be mapped on this motif node) is added to the motif iterator. The motif iterator will always first iterate over the set of nodes that can be mapped on the motif node that was last checked in the check list. Once it has iterated over all these network nodes, it will iterate further on the set of network nodes that can be mapped on the previous motif node according to the checklist. This explains the need of the checklist.

In the example the motif iterator (figure 6c) has an iterator over the set (of network nodes) {2, 10, 9, 7} for motif node 2 and an iterator over the set {12, 5, 3} for motif node 4. The other iterators are of no importance at this stage in the algorithm. At this moment network node 9 is mapped on motif node 2, which means that network nodes 2 and 10 were already mapped on motif node 2. Similarly network node 5 is mapped on motif node 4 meaning that network node 12 was already mapped on this motif node. When the algorithm continues, after finding network nodes that can be mapped on motif nodes 1 and 3, network node 3 will be mapped on motif node 3. When this iterator finishes, it is removed from the motif iterator and the search continues by mapping network node 7 on motif node 2.

Following functions are defined on an index iterator:

- put(motifnode, iterator): adds the iterator to the corresponding motifnode
- hasNext(): returns a boolean value indicating if any of the iterators has a next element. It will first check the iterator of the checklist.lastChecked(). If it is empty, it will check the iterator of the previous motif node. And so on.
- next(): returns the next element of the index iterator. This is the next element of the current motif node in the index list

#### Priorityqueuemap

The priorityqueuemap (PQM) is an instrument to determine the best possible motif node to be investigated next. It contains a priority queue for each motif node. Moreover, in order to know which of the motif nodes have not been investigated yet, a pointer to the checklist object is kept. This data structure is illustrated in figure 5c. It is similar to the motif iterator, but the iterators are substituted by priority queues (PQ).

In order to keep the search tree as small as possible, we want to select the (uninvestigated) motif node with the smallest set of possible network nodes that can be mapped on it. This set is the intersection of all the neighbor sets (a neighbor set is the set of all the network nodes that are connected to a mapped network node according to a specific link type) of nodes that can be mapped on this motif node. Since calculating this intersection can be time-consuming and the maximum cardinality of this intersection is the cardinality of the smallest of these neighbor sets, in the priority queues we will keep track of how many neighbors each mapped network node has according to the types of each of its outgoing links (according to the motif specification). By only taking into account the (cardinality of the) sets of neighbors of the mapped network nodes and not the intersection of these sets for one motif node, we do not produce the optimal (i.e. the smallest) search tree, but it is a good compromise between efficiency (calculating the intersection is time-consuming) and optimality.

The objects that are stored in the priority queues were designed specifically for the ISMA algorithm. We opted to name them priority objects (PO) (see figure 5d). Priority objects consist of 4 fields: a network node, a motif start node, a motif end node and the number of neighbors of the network node according to the type of the link between the motif start node and end node. The network node is the node of the current instance that has been mapped on the motif start node. A priority object contains 2 motif nodes, a start and an end node, from which the link type can be deduced. It should be noted that for one priority queue all the ‘motif end node’ fields are equal, and thus could be omitted. To determine the next best motif node, for each of the priority queues of the motif nodes that have not been chosen yet the minimum number of possible neighbors is retrieved. The overall minimum determines which motif node will be chosen next, as the maximum cardinality of the set of possible network nodes is minimal for this motif node.

In the example (see figure 6d) priority objects are indicated by a list of 4 elements ([network node, motif start node, motif end node, number of neighbors]). Here only the priority queues of motif node 1 and 3 are of importance. Each priority queue contains 2 priority object, one for each of the mapped network nodes. Motif node 3 would be selected as the best motif node to be investigated next, since network node 5 has the least number of neighbors that can be mapped on it.

The following functions are defined on a priorityqueuemap:

- add(priorityqueueObject): adds the priorityqueueObject to the correct priority queue according to its motif end node
- poll(): removes and returns the overall best element (i.e. priority object) of the priority queues

### The index-based subgraph matching algorithm (ISMA)

As mentioned previously, the recursive ISMA algorithm outperforms the naive recursive algorithm by always selecting the motif node with the smallest set of possible network nodes that can be mapped on it. In this way, the number of branches in the search tree is minimized.

Moreover, iterative algorithms can improve the performance and consume less stack space and function call overhead. In the previous section a number of data structures were presented in order to realize an iterative version of the ISMA algorithm. In this section the actual algorithm is discussed.

The pseudo code of the iterative ISMA algorithm is given below.

### Index-based Subgraph Matching Algorithm (ISMA)

1 findMotifs(mspec, Gt){

2  instance = EMPTY INSTANCE;

3  mn = determineFirstMotifNode();

4  CL.check(mn);

5  {startsetg = determineSet(instance, mn, Gt, mspec);

6  MI.put(mn, startset.iterator());

7

8  while(MI.hasNext()){

9   nn = MI.next();

10   backtrack();

11   instance.putNode(CL.lastChecked(), nn);

12   if(instance is complete){

13    export(instance);

14    continue on line 8;

15   }

16   forall(i in CL.rest()){

17    PQM.add(new PO(nn, CL.lastChecked(), i, #NBS));

18   }

19   mn = PQM.poll().getEndNode();

20   CL.check(mn);

21   set = determineSet(instance, mn, Gt);

22   MI.put(mn, set.iterator());

23  }

24 }

In the initialization phase (line 2–6) we will stipulate which of the motif nodes is best suited to be handled first (line 3). To determine this, for each link type present in the motif the number of occurrences in the network is counted. The start (motif) node of the link of the type with the least instances in the network is chosen to be the first motif node. If multiple motif nodes have an outgoing link of this type or multiple link types have the same number of instances, there are two options. One could randomly select one of these link types to determine the first motif node fast, but at the risk of creating a unnecessary branches in the search tree. The other option is to calculate the actual sets of candidate network nodes for these motif nodes by intersecting the sets for each of the link types departing from this motif node. Then the motif node with the smallest set of candidate network nodes is selected. This comes down to calculating the following intersection value (IV) for all motif nodes 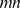 from which links of the specific types depart:
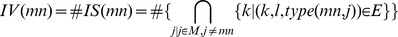
This is in fact the cardinality of the intersection (IS) of all sets of network start nodes of the links of the types that depart from 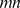. For 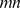 all types of the links departing from it are determined. For each of these link types the set of starting nodes in the network is collected and the cardinality of the intersection of all these sets is calculated. The minimal value (for the different motif nodes) of this parameter then determines which motif node will be handled first. The determination of the first motif node is heuristic in the sense that we want to find a good first motif node as soon as possible, while we cannot guarantee that the search tree we build is indeed the smallest one possible. Once it is known which motif node is the first to be investigated, a start set of network nodes is calculated (line 5). It consists of all network nodes that can be mapped onto this motif node. It should be noted that the determineSet-function is identical to the one used in the recursive ISMA algorithm. The chosen motif node is checked in the checklist (line 4), and an iterator over the determined start set is added to the motif iterator (line 6).

The main part of the algorithm executes the following as long as there are network nodes in the motif iterator (line 8). It retrieves the next network node from the motif iterator, and adds it to the instance on the position of the last checked motif node (line 9 and 11). If the instance is complete (i.e. all motif nodes have network nodes mapped on them), it is exported and the algorithm continues by retrieving the following network node from the motif iterator (line 12–15). If this is not the case, for all the motif nodes that have not been handled yet it is determined how many neighbors of this network node can be mapped on them and the results are added, in the form of a priority object, to the priorityqueuemap (line 16–18). Subsequently, the next best motif node is determined by retrieving the best priority object from the priorityqueuemap (line 19). This motif node is checked in the checklist, and an iterator over a set of network nodes is added to the motif iterator (line 20–22). This set is the result of the function determineSet that is identical to the one used in the recursive ISMA algorithm. For a complete description of this function we would like to refer to the section of the recursive algorithm.

The algorithm terminates when there are no more network nodes left in the motif iterator. This means that there are no more network nodes that are good candidates to be mapped on the motif nodes. All motif instances have thus been found.

It should be noted that, when the next motif node is retrieved from the motif iterator (line 9), the data structures are updated to allow backtracking. This is indicated by the backtrack-procedure (line 10). There are three possible situations. When a new iterator was added in the previous iteration, no updates are needed, and the algorithm can continue. If the motif iterator retrieves a motif node from the same iterator as in the previous iteration, both the motif instance and the priorityqueuemap need to be updated. The network node that was mapped in the previous iteration needs to be removed from the instance, so that a new network node can be mapped. Moreover, all priority objects that are associated with this previous network node need to be removed from the priorityqueuemap. If, on the other hand, the network node that is returned comes from an iterator that is associated with a motif node that was handled previously, all data structures are updated. In the checklist the motif nodes that have been investigated completely (i.e. the algorithm has iterated through all network nodes that can be mapped on them in the current situation) need to be unchecked. In the priorityqueuemap all priority objects that have these motif nodes as start node are removed. Moreover, all network nodes that are mapped on motif nodes of the rest-set of the checklist are removed from the instance, as well as the network node that is mapped on the current motif node (i.e. lastChecked()). The priority object associated with this last network node are also removed from the priorityqueumap.

### Dealing with symmetry

In this section it will be explained how the ISMA algorithm can be further optimized when dealing with symmetric motifs. A motif is called to be symmetric if it is identical to a motif from which the nodes are permutated in a certain way. The most common symmetries in motifs are reflections, rotations, translations and combinations of these three. By making use of the symmetry characteristics of a motif, the search tree of the (iterative) ISMA algorithm can be pruned further. Once a network node has been mapped on a motif node that takes part in a symmetric permutation, it should not be mapped again on the other nodes of this symmetry if this would lead to the same motif instance. In this paper, we will focus on two kind of symmetries, namely reflections (or mirror symmetry) and cyclic rotations, as these can easily be exploited to speed up the calculations. In the future, we plan to take into account all sorts of symmetries (like the algorithm of [27] does). At this time, for all other symmetries, duplicate instances will be eliminated once they have been found.

**Figure 7:**
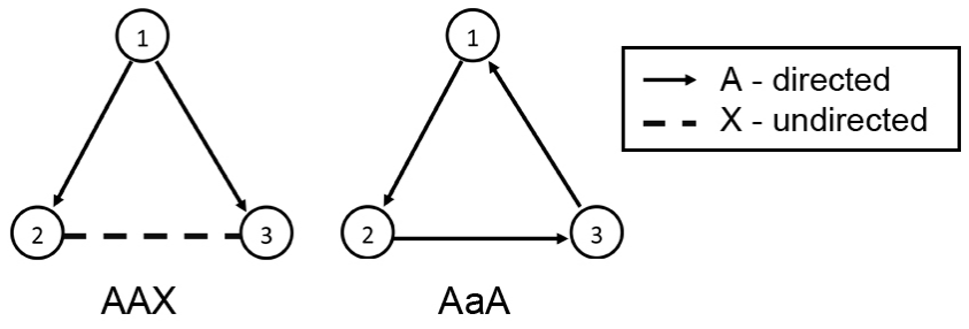
Examples of reflection and cyclic rotation symmetries. The motif on the left has a reflection symmetry between nodes 2 and 3. The motif on the right has a cyclic rotation symmetry between the three nodes.

A motif contains a reflection symmetry if and only if two or more nodes can be swapped without changing the motif's configuration. For example, the motif 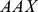 in figure 7 has a reflection symmetry between node 2 and node 3. A cyclic rotation symmetry is a symmetry in which nodes can be moved in circles. An example of a motif with this type of symmetry is 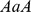 (figure 7) where the motif with nodes 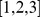 is equal to the motif with nodes 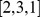 and the one with nodes 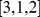.

**Figure 8:**
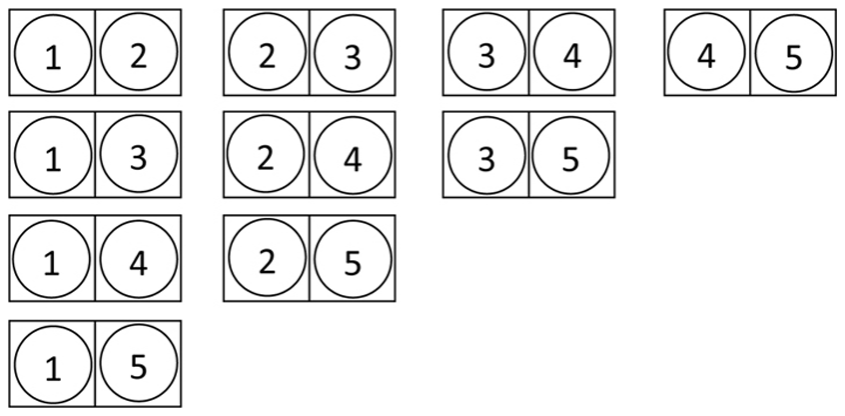
Reflection symmetry. Enumeration of all possibilities to map 5 network nodes on 2 reflection symmetric motif nodes. The squares represent motif nodes, the circles represent network nodes. Once a network node has been mapped on a motif node that is part of a reflection symmetry, it will never be mapped on one of the other nodes of the symmetry.

The idea behind ‘dealing with symmetry’ is that once we have mapped one network node on a motif node that is part of a symmetry, we do not want it mapped again on another motif node of the symmetry. Suppose that we have a reflection symmetry with 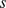 motif nodes and that there are 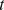 network nodes that can be mapped on them, then mapping the network nodes on these symmetric motif nodes comes down to choosing 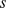 distinct elements out of a set of 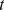 elements, not taking into account the order of the elements, which is in fact a combination of 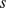 elements out of a set of 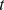 elements. One way to enumerate all these combinations is by first summing up all sets with the first element, then all sets with the second element that have not been encountered yet, etc. This is illustrated in figure 8. From this, it can be seen that, once one network node is mapped on a symmetric motif node (in the figure the first motif node), it will never be mapped again on one of the other symmetric motif nodes that are investigated thereafter.

**Figure 9:**
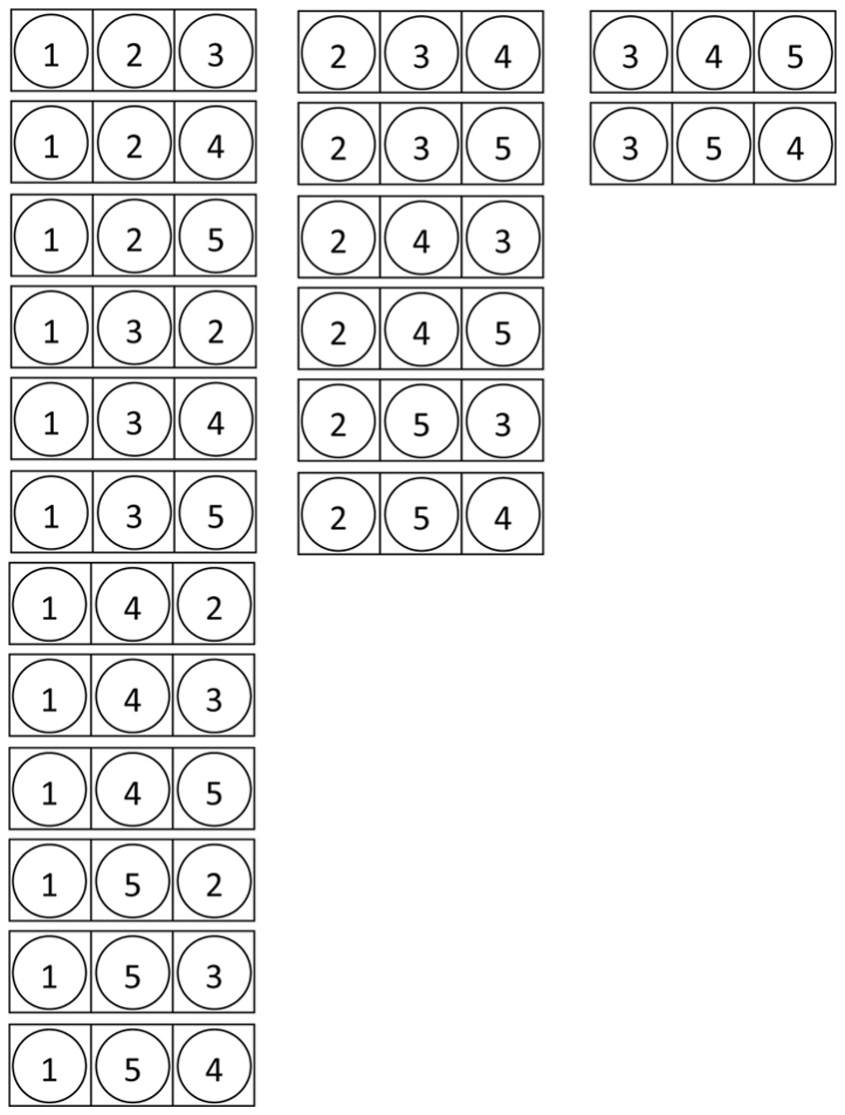
Cyclic rotation symmetry. Enumeration of all possibilities to map 5 network nodes on 3 motif nodes that are part of a cyclic rotation. The squares represent motif nodes, the circles represent network nodes. Once a network node is mapped on the ‘first’ motif of the symmetry, it will never be mapped on the other nodes of the symmetry. For the other motif nodes of the symmetry, all possibilities still need to be explored.

For cyclic rotation symmetries this is slightly different. Here one motif node needs to be chosen as the ‘first’ node of the rotation and the idea is that once a network node is mapped on this ‘first’ node, it cannot be mapped on one of the other motif nodes in the symmetry, while a network node that is mapped on the ‘second’ (or higher) motif node still could be mapped on the other nodes of the symmetry. This is illustrated in figure 9 where all possibilities are given to map 5 network nodes on 3 motif nodes that form a cyclic rotation. Once a network node has been mapped on the ‘first’ motif node of the rotation, it will not be mapped again on one of the other nodes in the rotation, while network nodes that are mapped on the ‘second’ motif node, still can be mapped on the ‘third’ motif node later on.

In order to realize a speedup by making use of these symmetry characteristics, some changes were made to the motif data structure and the algorithm. Additional information is stored in the motif, namely for each motif node a list is kept of the motif nodes with which it is symmetric. In the case of reflection symmetry, for each node of the symmetry this list contains all other nodes of the symmetry. In the case of cyclic rotations for the motif node, that has been chosen as the ‘first’ node, this list contains all other symmetric nodes, while the lists of the other motif nodes only contain one element, namely the ‘first’ motif node. Next to these changes in the motif definition, a novel data structure, called symmetry sets, was developed. For each symmetric motif node it contains a set of all possible network nodes that have not been mapped on it yet. These sets are used to help determining the new set of candidate network nodes (line 21 of the ISMA algorithm). If the motif node is symmetric, the (symmetry) sets of all nodes that are symmetric to it (according to the symmetry structure that was added in the motif definition), are added to the set of sets in the determineSet procedure. As these sets only contain the network nodes that have not been mapped yet, they make sure that all network nodes that have been mapped on symmetric motif nodes are eliminated when calculating the intersection.

In order to deal with symmetry (reflection or cyclic rotation) and further speed up the calculations, the ISMA algorithm is adapted in three ways:

- Every time it is determined which motif node will be investigated next (line 19), it is checked whether this node is part of a symmetry with motif nodes that have not been investigated yet. If this is the case, the set of network nodes that can be mapped on this motif node is added to the symmetry sets data structure. This is realized by adding following code after line 21.
  21a if(mn.isSymmetric()){
  21b symmetrySets.put(mn, set);
  21c }
- Every time a network node is retrieved from the motif iterator (line 9), the symmetry sets are updated. This means that, if this network node is mapped on a symmetric motif node, this network node is removed from the set associated with this motif mode. In this way it will not be mapped on the other motif nodes of the symmetry. This is realized by adding following code after line 11.
  11a if(CL.lastChecked().isSymmetric()){
  11b symmetrySets.remove(CL.lastChecked(), nn);
  11c }
- When the determineSet procedure is called, it is checked whether the motif node (i.e. index) is symmetric to nodes that have been investigated before. If this is the case, the corresponding (symmetry) sets are retrieved from the symmetry sets data structure and added to the set of sets, from which the intersection is calculated (line 55 of the recursive ISMA algorithm). This is realized by adding following code after line 54 in the determineSet-procedure.
  54a forall(mn in CL.checked()){
  54b if(mn.symmetricTo(motifnode)){
  54c  sets.add(symmetrySets.get(mn);
  54d }
  54e }

As mentioned before, the above adaptations narrow down the search tree of the ISMA algorithm by making use of the characteristics of the reflection and the cyclic rotation symmetries. Besides this, for all other types of symmetry, every time a new instance has been found, it will be checked whether it is symmetric to a previously found instance. If this is the case, the instance will not be exported (line 13). In order to check this, all motifs that are symmetric to this motif (except for reflections and cyclic rotations) need to be identified. For a motif with 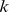 nodes, all symmetric motifs can be found by enumerating all permutations of the 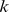 motif nodes, connecting them according to the motif specification, and comparing this newly formed motif to the original one. From this list of symmetric motifs, the reflection and cyclic rotation symmetries are eliminated, as they are already accounted for in the algorithm. By mapping the motif nodes of an instance to all symmetric motifs, it can easily be checked whether the instance is symmetric to a previous one.

In order to enumerate all permutations, a 17th century algorithm, called ‘plain changes’ by the English bell ringers, was used. In computer science, it is known as the Steinhaus-Johnson-Trotter algorithm [28]–[30], and it has been improved by Even [31]. It is a powerful algorithm that generates an ordering of all permutations and is able to find all 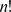 permutations of 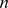 elements by swapping two adjacent elements 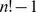 times. Due to the small differences between two consecutive permutations, this algorithm can be implemented in a constant time per permutation.

## Results and Discussion

This section starts with a description of the software that incorporates the index-based subgraph matching algorithm. Subsequently, a number of results are presented that indicate the strength of the algorithm in comparison with other subgraph matching algorithms.

### Software

A software implementation of the iterative ISMA algorithm is freely available at https://sourceforge.net/projects/isma/. It is presented in the form of a Java .jar -file (ISMA.jar) and can be used from a command line interface as follows:

java -jar “<directory>/ISMA.jar” -folder “<folder of input files>” -linkfiles “<list of <typename u/d filename> separated by spaces>” -motif “<motif>” -output “<reference to output file>”

The first two words indicate we want to execute a .jar-file. Subsequently, it is indicated where the .jar-file in question is situated. The program takes four arguments: folder, linkfiles, motif and output. The folder argument contains the directory where all input files are situated. It avoids retyping it for every inputfile. The linkfiles are the files that compose the network. Each linkfile contains all links for one specific type. It is denoted in the command by three arguments: the name of the link type (mostly an upper case character), a character indicating whether the links are directed (d) or undirected (u) and the name of the file. The different linkfiles are separated by spaces. The next argument is the motif. This is the motif specification as defined previously in this article. The last argument determines where the output should be stored.

The input files contain all links of one type. These links are represented by the name of the start node and the name of the end node separated by a tab. Every line contains one link. Example input files can be found online. The output file has one line for every motif that has been exported. A motif is represented as follows: Motif [<motifspecification>]: [<node 1>, <node 2>, …].

As the above notation is quite complicated, we will explain it in more detail by means of an example.

java -jar “ISMA/ISMA.jar” -folder “ISMA/input/”

-linkfiles “A d linksAtype.txt B d linksBtype.txt C u linksCtype.txt” -motif “ABC”

-output “ISMA/output/results.txt”

In this example ISMA.jar can be found in ISMA/. All input files are present in the folder ISMA/input/. The network contains links of three types: directed links of type A, directed links of type B and undirected links of type C. All these links can be found in the files linksAtype.txt, linksBtype.txt and linksCtype.txt respectively. The motif that is searched for is ABC, which is the motif we used in the example (see figure 3). The result of the ISMA algorithm (i.e. a list of all motifs found) is written to the file results.txt in the directory ISMA/output/.

In the ‘Files’ tab of this SourceForge project, all input (network) files that were used in the experiments are available. For a complete description of these networks, we would like to refer to the following section.

### Results

To demonstrate the strength of the ISMA algorithm, we compared it to the naive recursive subgraph matching algorithm (RSMA) as well as the algorithm of Ullmann [9], the VF algorithm [10], [11] and the VF2 algorithm [12], [13], which are state-of-the-art subgraph matching algorithms. We used two networks with multiple edge types as test networks. The first is an integrated network of physical (P, undirected), genetic (G, undirected) and signaling (S, directed) interactions between kinases and phosphatases in yeast [32], [33], also used in [26] (see left panel of Table 2 for basic network characteristics). The second consists of protein-protein interactions in yeast (X, undirected, obtained from the BioGRID [34] database), protein-protein interactions in human (Y, undirected, obtained from the BioGRID [34] and STRING [35] databases), and orthology relations between human and yeast proteins (Z, bipartite, from the InParanoid database [36]) (see right panel of Table 2 for basic network characteristics). Experiments were carried out on a 64-bit machine with a processor of the type Intel(R) Core(TM) 2 Duo CPU P8600, 2.40 GHz and 4 GB of RAM. Both the Recursive Subgraph Matching algorithm (RSMA) and the Index-based Subgraph Matching Algorithm (ISMA) were implemented in Java (version 1.6.0_18). The Ullmann, VF and VF2 algorithms are all present in the VFlibrary, a (sub)graph matching library implemented in C. These algorithms are known for finding subgraph isomorphisms in Attributed Relational Graphs (ARGs) fast, which is exactly what we are dealing with here.

**Table 2:** Network configurations of biological networks.

PGS network		XYZ network	
# nodes	1 255	# nodes	7 810
# S links	667	# X links	36 391
# G links	8 102	# Y links	40 630
# P links	3 688	# Z links	3 390

**Figure 10:**
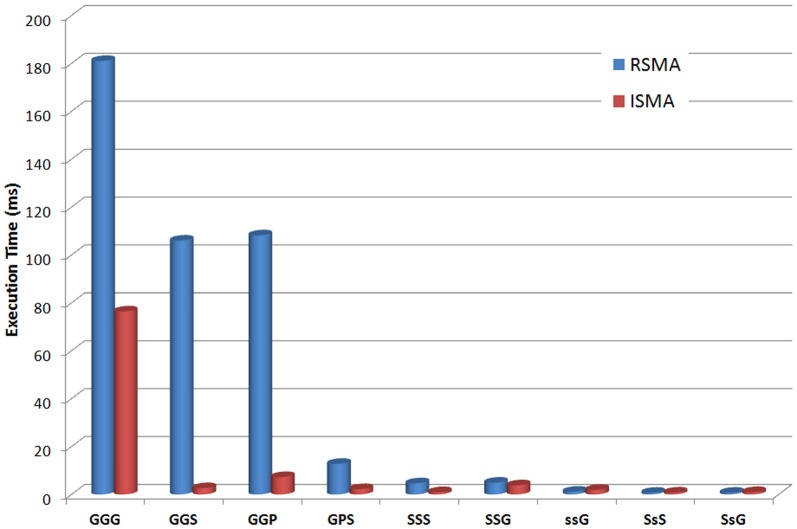
Execution times. Comparison of the execution times (in ms) of the RSMA and the ISMA algorithm for finding 3-node motifs in the PGS network.

We first searched for a number of three-node motifs in the PGS-network, that are useful in the clustering algorithm of [26], and monitored the execution time for each of the algorithms. In table 3 an overview is given of these execution times (in milliseconds). Here, it can be seen that there is a major difference between the algorithms of the VFlibrary and the (naive) RSMA and (iterative) ISMA algorithm. Despite the fact that they are implemented in C, which is a language with less execution overhead, the VFlibrary algorithms are remarkably slower. One of the decisive reasons for this discrepancy is the way how the network is stored in memory. For the ISMA and RSMA algorithm, the network structure has been optimized for fast retrieval of all start nodes of a certain link type (see Data Structures section). Looking at the RSMA and the ISMA algorithm separately (figure 10), we see that the ISMA algorithm indeed has lower execution times than the RSMA algorithm.

**Table 3:** The execution times (in milliseconds) for the different subgraph matching algorithms.

motif	# motifs	Ullmann	VF	VF2	RSMA	ISMA
GGG	9008	3653.5	4825.1	3421.1	181.0	76.4
SSS	81	851.0	145.6	80.4	4.6	0.9
GPS	47	1142.0	1061.9	769.4	12.8	2.3
SsS	3	815.2	140.3	76.7	0.8	0.8
SSG	103	861.4	155.7	87.0	4.9	3.8
SsG	31	838.4	145.5	83.0	1.0	1.0
GGS	312	1659.8	1167.6	866.7	106.0	2.6
GGP	418	1680.4	1201.5	898.7	108.2	7.3
ssG	112	868.2	163.6	94.5	1.9	1.9
8-clique	226	1 465 250	4 826 735	3 140 065	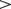 2 h	954
10-clique	1	4 759 462	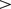 2 h	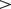 2 h	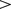 2 h	1 009
PGSPGS	0	1020.1	251.3	170.8	83.9	14.2
XZ00ZY	2558	535 657.0	111 610.0	42 514.0	153.7	93.9
XXXZ000Z0Y00ZYY	4745	1 828 683.0	825 828.0	182 734.0	2354.6	270.1
AAAZ000Z0B00ZBB	840	726 732.0	166 608.0	34 053.0	595.2	123.2

It should be noted that the experiments were interrupted after 2 hours.

Subsequently, we looked for a number of larger motifs in this network. Table 3 shows the execution times of the algorithms when searching for all 8-cliques and 10-cliques (i.e. complete graphs of 8 and 10 nodes respectively). While for the ISMA algorithm, the execution times are around 1 second, they run up to more than two hours for the other algorithms. Here, the RSMA algorithm even performs worse that the algorithms of the VFlibrary. It should be noted that the difference between the ISMA algorithm and the other algorithms is most extreme for ‘complete’ motifs (i.e. cliques). However, for sparser motifs still significant speedups are realized, as will be shown in the experiments with the XYZ-network.

Moreover, we looked for a motif that is not present in the PGS-network, viz the 4-node motif PGSPGS. Results are shown in table 3. While the execution times are relatively low, the difference between the algorithms of the VFlibrary and the RSMA and ISMA algorithms is not as remarkable as in the previous cases.

Next we searched in the XYZ-network for so-called *interologs*, 4-node subgraphs (with motif specification XZ00ZY) consisting of conserved protein-protein interactions between orthologous protein pairs in yeast and human [37]. Generalizing this concept, we also searched for conserved triangles (6 nodes, motif specification XXXZ000Z0Y00ZYY). Despite of the fact that protein-protein interactions are undirected, in the experiments we also assumed the links to be directed. The directions were determined by the input files as the first and the second proteins were considered tails and heads respectively. In this directed network, we looked for the 6-node motif AAAZ000Z0B00ZBB. Here as well, we observe dramatic reduction in execution times for ISMA (and to a lesser extent RSMA) compared to the VF library algorithms, as these tend to be quite slow for larger motifs.

In conclusion, by an intelligent design of the network data structure a remarkable speedup is realized for the RSMA and ISMA algorithm in comparison to the VFlibrary algorithms. Moreover, this speedup is increased even more by carefully selecting the order in which the motif nodes are investigated (i.e. ISMA vs. RSMA).

To quantify the relative speedup realized by the ISMA algorithm, we defined the calculation time multiplicator (CTM) as
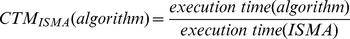
For clarity reasons, in the remainder of this article we will use 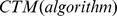 in stead of 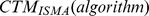.

These calculation time multiplicators are given in table 4. It shows that the highest speedup factors are achieved for the motifs with an average number of occurrences. These figures show that the larger the motif, the larger the speedup that can be realized (XZ00ZY vs. XXXZ000Z0Y00ZYY) and that these speedups are higher when there are more occurrences in the network (XXXZ000Z0Y00ZYY vs. AAAZ000Z0B00ZBB). In conclusion, 2 factors contribute in reducing the calculation type: the network data structure that has been optimized for fast retrieval of all links of a certain type and the order in which the motif nodes are investigated in order to reduce the search tree. While the former one explains the difference between the VFlibrary algorithms and the RSMA and ISMA algorithm, the latter one causes the execution time of the ISMA algorithm to be smaller than that of the RSMA algorithm.

**Table 4:** The calculation time multiplicators (CTM) of the ISMA algorithm compared to other algorithms for a number of motif configurations.

motif	# motifs	CTM(Ullmann)	CTM(VF)	CTM(VF2)	CTM(RSMA)
GGG	9008	47.8	63.1	44.8	2.4
SSS	81	873.7	149.5	82.5	4.8
GPS	47	505.5	470.1	340.6	5.7
SsS	3	1000.2	172.1	94.1	1.0
SSG	103	225.0	40.7	22.7	1.3
SsG	31	831.7	144.3	82.3	1.0
GGS	312	630.6	443.6	329.3	40.3
GGP	418	231.2	165.3	123.6	14.9
ssG	112	457.4	86.2	49.8	1.0
8-clique	226	1 535.9	5 059.5	3 291.5	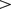 7 547.2
10-clique	1	4 717.0	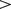 7135.8	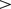 7 135.8	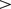 7 135.8
PGSPGS	0	72.1	17.8	12.1	5.9
XZ00ZY	2558	5704.5	1188.6	452.8	1.7
XXXZ000Z0Y00ZYY	4745	6771.3	3057.9	676.6	8.7
AAAZ000Z0B00ZBB	840	5900.3	1352.7	276.5	4.8

**Figure 11:**
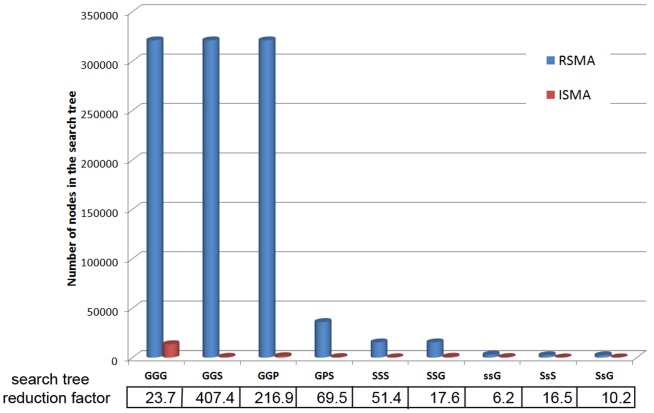
Size search tree. Comparison of the number of nodes in the search tree of the RSMA and the ISMA algorithm for finding 3-node motifs in the PGS network. The search tree reduction factor is defined as the size of the search tree of RSMA divided by the size of the search tree of ISMA.

As explained in the Methods section, the ISMA algorithm achieves its speedup compared to the RSMA algorithm by reducing the size of the search tree. We counted the number of nodes in the search tree for both algorithms when searching for 3-node motifs in the PGS-network (see figure 11). On average, the search trees of the ISMA algorithm are around 100 times smaller than the corresponding search trees of the RSMA algorithm. It should be noted that the size of the search tree (and the execution time) is dependent on both the motif configuration and the network in which these motifs are searched for.

Although the development of the ISMA algorithm was motivated by the problem of identifying composite motifs in biological networks with multiple interaction types, or more generally Attributed Relational Graphs, it can of course be applied equally well to non-biological networks. We illustrate this by searching for all 3-node cliques in a number of networks from the SNAP database (http://snap.stanford.edu/data/index.html), where we treated all networks as undirected. Assuming all link are of the type X, this means the motif XXX is searched. Table 5 shows the configurations of the networks that were used in the experiments, together with the number of 3-node cliques that were found in these networks. In table 6 the CTM's are given of the ISMA algorithm over the algorithms of the VFlibrary and over the recursive algorithm. Moreover, the search tree reduction factors (i.e. the number of nodes in the search tree of RSMA divided by the number of nodes in the search tree of ISMA) are depicted. This table shows that, similar to the experiments on biological networks, the execution times of the algorithm presented in this article are much lower that those of the algorithms of the VFlibrary. Again, this can be explained by the network data structure that allows fast retrieval of all links of a certain type. If we take into account the large numbers of (XXX) motifs that are present in these networks, the CTM's are relatively small in comparison to those in biological networks. This is also confirmed in the search tree reduction factors. Here the search tree reduction factors are on average around 20, which is small in comparison with the average search tree reduction factor of around 100 for the biological networks. The reason for this is that here, when determining the set of network nodes that can be mapped on a motif node, all neighbors of the mapped network nodes need to be taken into account in stead of only the neighbors according to one specific link type.

**Table 5:** Network configurations of the non-biological networks, together with the number of 3-node cliques present in these networks.

network	# nodes	# links	# motifs
Wiki-Vote	7115	103689	608389
p2p-Gnutella08	6301	20777	2383
p2p-Gnutella30	36682	88328	1590
CA-CondMat	23133	186936	173361
CA-HepTh	9877	51971	28339

**Table 6:** The calculation time multiplicators (CTM) of the ISMA algorithm compared to other algorithms for a number of non-biological networks.

network	CTM(Ullmann)	CTM(VF)	CTM(VF2)	CTM(RSMA)	STRF
Wiki-Vote	126.8	211.9	142.8	11.1	41.2
p2p-Gnutella08	131.9	27.3	12.9	1.6	26.5
p2p-Gnutella30	706.4	35.7	6.4	1.1	17.7
CA-CondMat	870.1	565.2	241.3	2.3	15.0
CA-HepTh	293.4	195.0	83.5	1.5	11.1

The last column shows the search tree reduction factors (STRF), i.e. the ratio of the number of nodes in the search tree of the RSMA algorithm to the search tree of the ISMA algorithm.

### Conclusion

Motivated by problems in the analysis of biological networks composed of multiple directed and undirected interaction types, we have developed a novel exact subgraph matching algorithm that is optimized for graphs with specific link characteristics. By carefully selecting the order in which the nodes of a network motif are investigated and by designing appropriate data structures, a remarkable speedup can be realized. In each iteration of the algorithm, sets of network nodes are determined that can be mapped on the remaining motif nodes. Always selecting the motif node with the smallest corresponding set of network nodes leads to less branches closer to the root of the search tree and consequently a reduced search tree.

In order to realize an iterative version of this algorithm, a number of data structures were developed: a checklist that keeps track of the order in which the motif nodes are investigated, a motif iterator to iterate over all the network nodes that can be mapped on a motif node, and a priorityqueuemap in order to select the best motif node to be investigated next.

Incorporating motif symmetries can lead to further increases in computational efficiency. When present, ISMA explicitly takes into account two specific symmetries, namely the reflections and cyclic rotations, to further speed up the algorithm. For all other motif symmetries, duplicate instances are eliminated once they have been created. In future versions of ISMA, we plan to take into account additional types of symmetries to prune the search tree.

Applications on real network data from the biological as well as non-biological domain, showed that the ISMA algorithm indeed leads to speedups compared to existing exact subgraph matching algorithms for attributed relational graphs. A comparison with a naive recursive tree-based subgraph matching algorithm shows that to a large extent, this speedup is indeed due to tree-pruning strategy implemented in ISMA, with search trees in ISMA being on average 100 times smaller than those of the recursive algorithm in our experiments on biological networks and on average 20 times smaller in our experiments on non-biological networks.

Taken together, we believe ISMA is an interesting new exact subgraph matching algorithm which will be important for the discovery and analysis of small and large network motifs in ever growing biological networks, with potential applications in other domains as well.
